# Direct comparison of distinct naive pluripotent states in human embryonic stem cells

**DOI:** 10.1038/ncomms15055

**Published:** 2017-04-21

**Authors:** S. Warrier, M. Van der Jeught, G. Duggal, L. Tilleman, E. Sutherland, J. Taelman, M. Popovic, S. Lierman, S. Chuva De Sousa Lopes, A. Van Soom, L. Peelman, F. Van Nieuwerburgh, D. I. M. De Coninck, B. Menten, P. Mestdagh, J. Van de Sompele, D. Deforce, P. De Sutter, B. Heindryckx

**Affiliations:** 1 Department for Reproductive Medicine, Ghent-Fertility and Stem cell Team (G-FaST), Ghent University Hospital, 9000 Ghent, Belgium; 2 Nuffield Department of Clinical Neurosciences, John Radcliffe Hospital, University of Oxford, Oxford OX3 9DS, UK; 3 Department of Pharmaceutics, Laboratory for Pharmaceutical Biotechnology, Faculty of Pharmaceutical Sciences, Ghent University, 9000 Ghent, Belgium; 4 Department of Anatomy and Embryology, Leiden University Medical Center, 2333 ZA Leiden, The Netherlands; 5 Department of Reproduction, Obstetrics and Herd Health, Faculty of Veterinary Medicine, Ghent University, 9820 Merelbeke, Belgium; 6 Department of Nutrition, Genetics and Ethology, Faculty of Veterinary Medicine, Ghent University, 9820 Merelbeke, Belgium; 7 Department of Pediatrics and Medical Genetics, Center for Medical Genetics, Ghent University Hospital, 9000 Ghent, Belgium

## Abstract

Until recently, human embryonic stem cells (hESCs) were shown to exist in a state of primed pluripotency, while mouse embryonic stem cells (mESCs) display a naive or primed pluripotent state. Here we show the rapid conversion of in-house-derived primed hESCs on mouse embryonic feeder layer (MEF) to a naive state within 5–6 days in naive conversion media (NCM-MEF), 6–10 days in naive human stem cell media (NHSM-MEF) and 14–20 days using the reverse-toggle protocol (RT-MEF). We further observe enhanced unbiased lineage-specific differentiation potential of naive hESCs converted in NCM-MEF, however, all naive hESCs fail to differentiate towards functional cell types. RNA-seq analysis reveals a divergent role of PI3K/AKT/mTORC signalling, specifically of the mTORC2 subunit, in the different naive hESCs. Overall, we demonstrate a direct evaluation of several naive culture conditions performed in the same laboratory, thereby contributing to an unbiased, more in-depth understanding of different naive hESCs.

## Abstract (web summary)

Human embryonic stem cells (hESCs) in culture display a state of primed pluripotency, but recent protocols have been developed that enable hESCs to adopt a naive-like pluripotent state. Here the authors perform a side-by-side comparison of methods used to culture naive hESCs and confirm the role of PI3K/AKT/mTORC signalling in facilitating the induction of naive pluripotency.

Although both originate from the inner cell mass of pre-implantation blastocysts^1^,^2^, mouse embryonic stem cells (mESCs) and human ESCs (hESCs) exhibit distinctive characteristics. Human ESCs display a primed state of pluripotency, similar to mouse epiblast stem cells (EpiSCs)^3^,^4^ derived from the post-implantation epiblast. Primed hESCs display flat colony morphology, low single-cell clonogenicity, dependence on TGFβ/activin/nodal signalling and show inefficiency to contribute to chimeras. Conversely, mESCs reside in the so-called naive pluripotent state, characterized by domed colonies, increased single-cell survival, dependence on JAK/STAT signalling and efficient contribution to chimeras^5^,^6^,^7^. Their high single-cell clonogenicity facilitates bulk culture, deeming them more favourable for future practical applications. Moreover, naive mESCs are also more homogeneous, leading to unbiased and efficient directed differentiation towards germ layer derivatives^8^,^9^. Recently, several groups have formulated protocols to induce naive pluripotency in hESC, both from pre-existing primed hESCs and by direct derivation from the blastocyst stage. This has been achieved using naive human stem cell media (NHSM)^10^, the reverse-toggle (RT) protocol^11^, 5i/L/F/A medium^12^ or by ‘resetting' primed hESCs, via ectopic *NANOG* and *KLF2* expression, followed by exposure to naive culture medium without bFGF^13^. Likewise, our group recently formulated a novel naive conversion medium (NCM) that facilitates the induction of naive pluripotency from primed hESCs, as well as the derivation of naive mESCs from the blastocyst stage^8^. To date, efforts to compare naive hESC induced via different culture conditions have primarily been performed using published data sets from several groups, which may introduce bias due to the use of different stem cell lines in different laboratories and different transcriptomic platforms employed. Takashima *et al*.^13^ hybridized their reset cells on a similar microarray platform as NHSM-naive hESCs and compared their biological results with published data sets for NHSM and 3iL-naive hESCs. Lineage-specific markers, such as *AFP* and *EOMES* were expressed in NHSM and 3iL-naive hESCs, while chromatin modifiers were downregulated compared to reset cells^13^. Whether these findings are truly biologically representative is speculative as only the reset cells were induced in culture, while the analysis of NHSM and 3iL-naive hESCs exclusively relied on published data sets from other studies. In addition, several differences were observed between the different lines converted in NHSM conditions^13^. Further, detailed comparative computational analysis of naive and primed states^14^ also suggested that the resulting differentially expressed genes are taken as a proxy of the target protein, which may lead to false positive and false negative results^14^. Therefore, to achieve more comprehensive and reliable biological conclusions, performing experiments in parallel within the same laboratory and on identical hESC lines, rather than solely relying on data set analysis, is imperative. An alternative study performed weighted gene co-expression network analysis on various naive data sets^15^ and suggested a potential overlap between reset cells and 5iL/F/A-naive hESCs with minor overlap with primed hESCs. However, the authors stress for caution in data interpretation, as the variations in culture conditions result in transcriptomic noise. Therefore, in order to accurately evaluate biological differences, culture conditions require standardization, while the transcriptomic sample numbers need to be increased^15^. Hence, we report, a detailed comparative analysis of naive hESCs converted in distinct naive media conditions in the same laboratory to provide a more unbiased approach to study the underlying variances between these differentially converted naive hESCs. Since all naive culture conditions to convert primed state hESCs towards a naive pluripotent state used mouse embryonic fibroblast (MEFs) as feeder layer, we have denoted the three conditions as NCM-MEF, NHSM-MEF and RT-MEF below. We report efficient conversion of three in-house-derived primed hESCs towards a naive pluripotent state, specifically in NCM-MEF and NHSM-MEF media. We also demonstrate that the naive hESCs exhibit dependence on mTORC2 subunit of PI3K/AKT/mTORC pathway, are unique transcriptomically to their parental primed counterparts and can differentiate efficiently towards lineage-specific progenitors.

## Results

### Enhanced naive state displayed by NCM/NHSM-MEF naive hESCs

To study the characteristics and properties of differentially induced naive hESCs in a controlled setting, we converted existing, in-house-derived three primed hESC lines, within the same laboratory, in different culture conditions. These included NHSM, RT and the NCM protocol. We specifically selected these protocols, as they were shown to generate karyotypically normal naive hESCs, without the need for gene transfection. We also selected conversion on MEF feeder layer considering only NHSM culture medium is thus far capable of supporting feeder-free culture of naive hESC. Hence, to avoid bias, we utilized standard naive culture conditions on MEF feeder layer for all three naive media for downstream comparative analysis, similar to primed hESC culture conditions.^10^. Although, secondary optimization also revealed PKCi and ROCKi as optional boosters for NHSM-naive hESCs condition^10^ and ROCKi for NCM^8^, we only selected the initial core components of each media for the comparative analysis on MEF feeder layer. The naive hESC lines induced in 5i/L/F/A medium were shown to be karyotypically abnormal and exhibited irreversible loss of imprinting^12^,^16^, which is concerning as their inability of methylation memory inheritance can influence differentiation capacity for future therapeutic applications. As well, the ‘reset cells' require ectopic expression of *NANOG* and *KLF2,* prior to culture in naive culture medium, and thus, these naive culture conditions were excluded from this study^13^.

**Figure 1:**
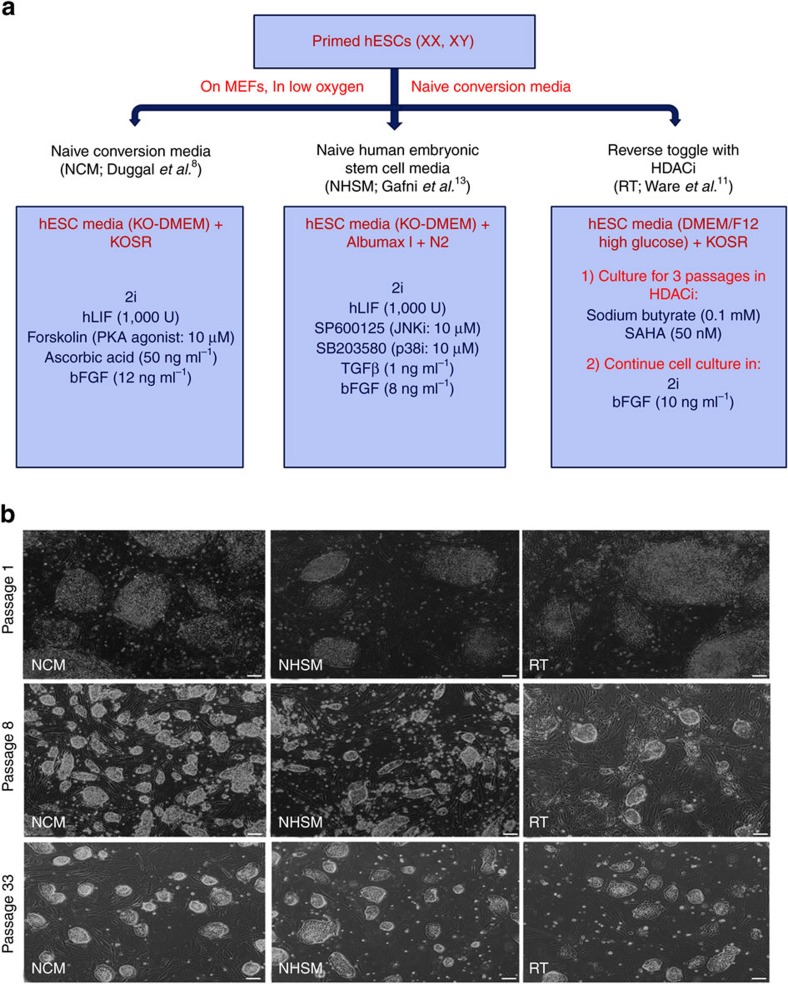
Conversion of primed hESCs towards a naive state in different naive media combinations. (**a**) Overview of small molecules and growth factors supplemented in NCM, NHSM and RT naive media. (**b**) Morphological analysis of hESC cultures in different naive media and their transition from primed pluripotency-specific flat morphology towards dome-shaped undifferentiated naive colonies. NCM and NHSM media comprised of several domed colonies whereas their frequency was lower in RT media. Scale bars, 200 μm.

**Figure 2:**
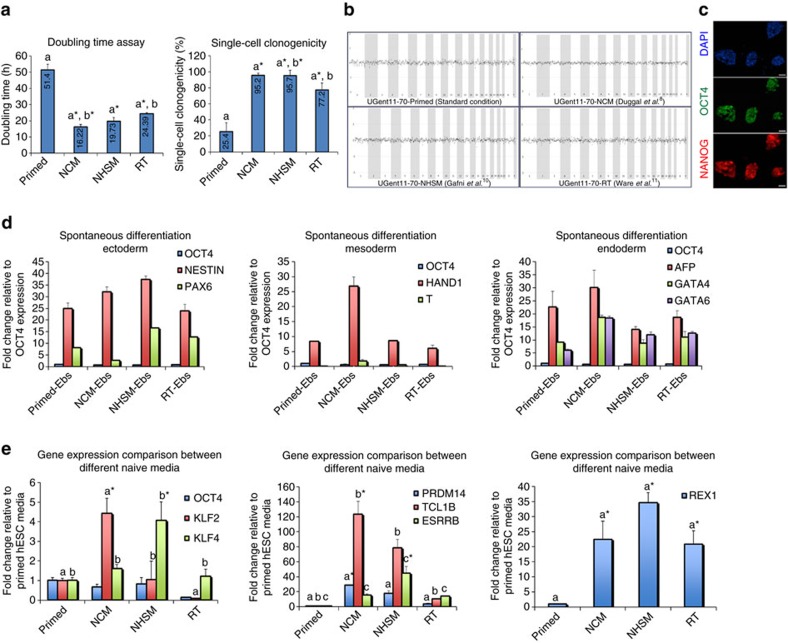
Assessment of naive pluripotency in the newly formed naive hESCs from primed hESC line UGent11-70. (**a**) Doubling time and single-cell clonogenicity assays for primed hESCs, NCM-, NHSM- and RT-naive hESCs. a* compared to a and b* compared to b, *P<0.05. (**b**) Depiction of chromosomal analysis for primed hESCs, NCM-, NHSM- and RT-naive hESCs via array comparative genome hybridization (aCGH). (**c**) Representative immunofluorescence image for pluripotency makers OCT4 and NANOG in NCM-, NHSM- and RT-naive hESCs; Scale bars, 200 μm. (**d**) qRT-PCR analysis for ectoderm, mesoderm and endoderm markers on spontaneously differentiated primed hESCs, NCM-, NHSM- and RT-naive hESCs as EBs for 14 days. (**e**) Gene expression analysis for naive-pluripotency specific genes on undifferentiated primed hESCs, NCM-, NHSM- and RT-naive hESCs. a* compared to a and b* compared to b, *P<0.05. The data are represented as mean±s.d.

Upon exposing the in-house-derived primed hESC lines in NCM-MEF, NHSM-MEF and RT-MEF conditions (Fig. 1a), we observed successful conversion towards a more naive state in all three conditions, with the first evidence depicted by the appearance of domed-like colony morphology (Fig. 1b). These mouse-like naive colonies appeared within 5–6 days in NCM-MEF, 6–10 days in NHSM-MEF and 14–20 days in RT-MEF naive media. NCM-MEF cells manifested a faster doubling time (12–18 h) compared to NHSM-MEF (14–19 h) and RT-MEF (22–24 h; Fig. 2a, Supplementary Figs 1 and 2). Similarly, NCM-MEF and NHSM-MEF naive hESC lines demonstrated high single-cell survival (95%), whereas RT-MEF naive hESC lines showed a lower efficiency of 77%. Nonetheless, the doubling time and single-cell clonogenicity of the cells converted in all naive media continued to be significantly variable (*P*<0.05) compared to their primed counterparts, with primed hESC lines exhibiting increased doubling time of 47–52 h and decreased single-cell clonogenicity of only 25% (Fig. 2a, Supplementary Figs 1 and 2). Next, all primed hESCs and their NCM-MEF naive counterparts exhibited a normal chromosomal profile as verified by array comparative genome hybridization, however, in NHSM-MEF and RT-MEF naive hESC culture conditions, only UGent11-70 and UGent11-60/UGent11-70 naive hESCs were karyotypically normal, respectively (Fig. 2b, Supplementary Figs 1 and 2). Converted naive hESCs were positive for OCT4/NANOG (Fig. 2c, Supplementary Figs 1 and 2) and spontaneously differentiated into all the three germ layers (Fig. 2d, Supplementary Figs 1 and 2). Interestingly, embryoid bodies (EBs) derived from NCM-MEF naive hESCs showed a consistent differentiation efficiency towards ectoderm (*NESTIN*), mesoderm (*HAND1*) and endoderm (*AFP, GATA4, GATA6*). EBs from NHSM-MEF naive hESCs expressed lower levels of *HAND1* and those from RT-MEF naive hESCs showed decreased expression of both *NESTIN* and *HAND1* (Fig. 2d).

### Naive hESCs rely on mTORC2 subunit to maintain pluripotency

We further confirmed naive pluripotency by quantitative real-time PCR (qRT-PCR) gene expression analysis on the three naive hESC lines and their primed counterparts. Significantly increased *KLF2* (*n*=3, *P*<0.05) expression was observed in NCM-MEF naive hESCs, while *KLF4* (*n*=3, *P*<0.05) was expressed higher in NHSM-MEF naive hESCs. RT-MEF naive hESCs showed reduced expression of these markers, similar to primed hESCs. *OCT4* expression was consistent in all hESC lines (Fig. 2e). The naive marker *REX1* was highly expressed in all naive hESC lines compared to primed (*n*=3, *P*<0.05), however, lower expression observed in RT-MEF naive hESC lines compared to NCM- and NHSM-MEF naive hESC lines (Fig. 2e, Supplementary Figs 1 and 2).

We observed a significant upregulation of naive markers including *KLF2* (6–10-fold), *PRDM14* (5–40-fold) and *TCL1B* (60–80-fold) in hESCs converted in the NCM-MEF protocol compared to NHSM- and the RT-MEF protocol (*n*=3, *P*<0.05, Fig. 2e, Supplementary Figs 1 and 2). This suggests that in NCM-MEF naive hESCs, *KLF2* and *PRDM14* (a PR-domain-containing transcriptional regulator), both known to work synergistically to facilitate the reprogramming of EpiSCs towards a naive mESC-like state of pluripotency^17^,^18^,^19^,^20^ have a similar role in inducing naive pluripotency in hESCs. Interestingly, NHSM-MEF naive hESCs continued to show significantly increased expression of *ESRRB* (*n*=3, *P*<0.05) compared to NCM- and RT-MEF naive hESCs (Fig. 2e, Supplementary Figs 1 and 2). This correlates with their elevated *KLF4* expression level (Fig. 2e Supplementary Figs 1 and 2), as *ESRRB* is a direct target of *KLF4,* as seen in mESCs both also being downstream targets of *NANOG* (refs 21, 22). The activation of the LIFRβ/gp130 receptor by LIF can activate the PI3K pathway, which acts on the serine/threonine protein kinase B (PKB or AKT) downstream effectors^23^. *TCL1B* is known to enhance the AKT kinase activity and induce naive pluripotency^24^. This is further facilitated by *PRDM14*, which inhibits FGF signalling and activates AKT-mTORC signalling^17^,^18^,^19^. This was further confirmed upon exposing primed, NCM-MEF, NHSM-MEF, RT-MEF naive hESCs to rapamycin (mTORC1 inhibitor)^25^ and PP242 (mTORC1/2 inhibitor)^26^ (Supplementary Fig. 3). Compared to primed hESCs, all naive hESCs exhibited increased apoptosis in response to rapamycin treatment, which increased in PP242 condition (Supplementary Fig. 3). Similarly, NANOG expression was almost absent in PP242-treated naive hESCs whereas rapamycin-treated naive hESCs showed marked downregulation in NANOG levels (Supplementary Fig. 3). Hence, as naive hESCs express increased levels of *PRDM14* and *TCL1B* in contrast to primed hESCs, albeit at varying levels, and undergo apoptosis upon exposure to mTORC inhibitors (especially mTORC2 inhibitor), our results suggest an intriguing role of the PI3K/AKT/mTORC-signalling pathway in facilitating the naive state in hESCs. This is supported by the knowledge that the self-renewal of naive mESCs is dependent on PI3K/AKT, LIF/STAT3, BMP/SMAD signalling which work together to inhibit primed pluripotency-associated ERK and GSK3β pathways, thereby allowing *Nanog* and *c-Myc* to maintain pluripotency^27^,^28^. Importance of mTORC has been highlighted in naive mESCs and mouse embryos whereby, disruption of mTORC resulted in their reduced cell size as well as impaired proliferation^29^. High levels of bFGF also activate the PI3K/AKT/mTOR pathway, leading to the inhibition of MAPK/ERK (MEK) signalling essential for the naive pluripotent state while low FGF continues to activate the MEK pathway^30^. Hence, increased concentration of bFGF (8-12 ng ml^−1^) in all naive media directly relates to the upregulation of PI3K/AKT/mTOR and suppression of the MEK pathway in naive hESCs. This is contrary to the primed state, in which MEK signalling is maintained via PI3K/AKT signalling, due to the lower FGF concentration (4 ng ml^−1^)^31^. To date, only one group has established naive hESCs in the absence of bFGF^13^, hence the role of bFGF in the establishment and maintenance of naive hESC remains to be elucidated.

### Naive hESCs differentiate robustly towards immature cell types

**Figure 3:**
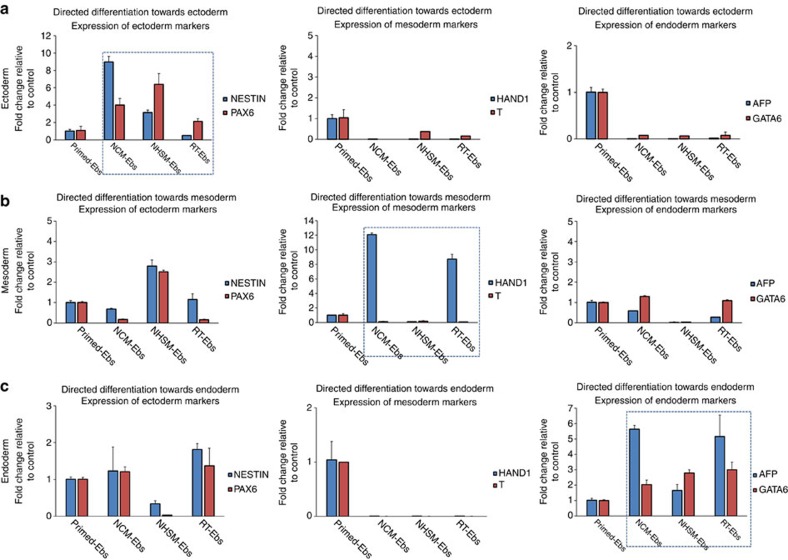
Germ-layer specific differentiation of naive hESC line UGent11-70. Gene expression analysis for lineage-specific genes upon directed differentiation of naive hESC line UGent11-70 towards (**a**) ectoderm, (**b**) mesoderm and (**c**) endoderm. All germ layer markers were analysed irrespective of the targeted lineage to determine the heterogeneity in differentiation between the different naive medium conditions as well as between primed and naive conditions. Dashed box represents the gene expression for markers specific for that lineage. The data are represented as mean±s.d.

**Figure 4:**
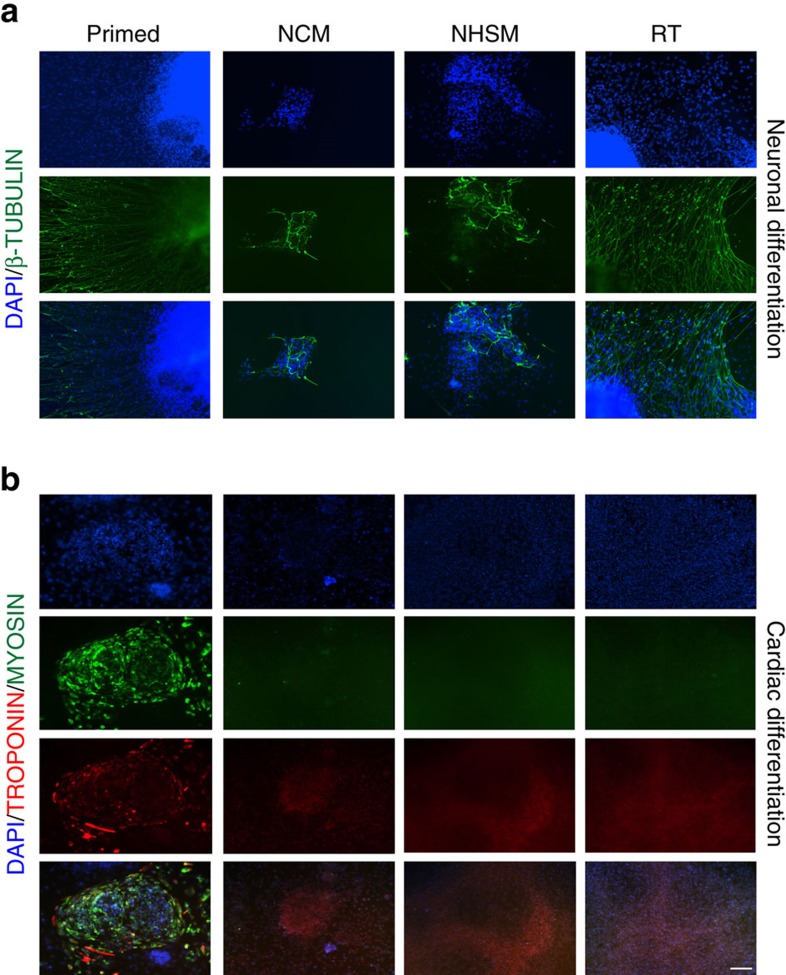
Directed differentiation of primed and naive hESCs towards neuronal and cardiac lineages. (**a**) Representative images of primed, NCM-, NHSM- and RT-MEF naive hESCs expressing β-TUBULIN when differentiated towards neuronal lineage. β-TUBULIN is expressed higher in neurons derived from primed and RT-MEF naive hESCs compared to NCM- and NHSM-MEF naive hESCs. (**b**) Representative images of primed, NCM-, NHSM- and RT-MEF naive hESCs expressing mature cardiac markers TROPONIN and MYOSIN when differentiated towards cardiomyocytes. Primed hESCs when differentiated towards cardiac lineage expressing markers TROPONIN and MYOSIN whereas all naive hESCs fail to express the mature markers. Scale bar, 200 μm.

Further, we aimed to study the lineage-specific differentiation potential of naive hESCs. Upon exposing the EBs derived from NCM-, NHSM- and RT-MEF naive hESCs to ectodermal differentiation cues, we observed high upregulation of *NESTIN* and *PAX6,* by 8- and 6-fold (*n*=3, *P*<0.05) in NCM and NHSM-EBs, respectively, compared to RT-EBs and their primed counterparts (Fig. 3a, Supplementary Figs 4 and 5). We observed no expression of mesodermal or endodermal markers in all naive-EBs, whereas EBs from primed hESCs continued to express markers of all lineages, albeit at low levels. This indicates that naive hESCs are transcriptionally more homogenous, similar to mESCs^7^,^9^. When subjected to mesoderm-directed differentiation, NCM-EBs exhibited highly efficient differentiation, with *HAND1* significantly upregulated by 10-fold (*n*=3, *P*<0.05) compared to NHSM-EBs, which showed lower potential to differentiate towards mesoderm and continued to express ectoderm genes. RT-EBs also exhibited efficient mesoderm differentiation, as depicted by a 2 to 8-fold increase in *HAND1* expression compared to NHSM-EBs and primed-EBS (Fig. 3b, Supplementary Figs 4 and 5). Finally, NCM-EBs, NHSM-EBs and RT-EBs underwent robust endoderm-directed differentiation indicated by increase in *AFP* and *GATA6* expression levels (*n*=3, *P*<0.05), while primed hESC-EBs continued to express ecto and mesodermal markers (Fig. 3c, Supplementary Figs 4 and 5). To study the functional capacity of these converted naive hESCs compared to their primed origins, we induced neuronal^32^ and cardiac differentiation^33^ using well-established protocols for hESC. Differentiation towards the neuronal lineage facilitated the induction of neuronal rosettes for all conditions, although only primed hESCs formed well-characterized and recognizable neuronal processes (Supplementary Fig. 6a). For cardiac differentiation, spontaneously beating population of cells were only observed in cells differentiated from primed hESCs (Supplementary Fig. 6b). Although both naive and primed hESCs expressed early neuronal marker PAX6 and cardiac progenitor markers NKX2.5/GATA4 (Supplementary Figs 7,8), only differentiated primed hESCs (and to some extent RT-naive hESCs) expressed the mature neuronal markers β-TUBULIN and TROPONIN/MYOSIN for mature cardiac lineage (Fig. 4). This supports the absence of spontaneously beating clusters in cardiomyocytes differentiated from naive hESCs, in contrast to primed hESC where beating clusters were observed.

Overall, although we see upregulation of lineage-specific markers for naive hESCs, their ability to undergo terminal differentiation towards functional cell types is limited compared to primed hESCs in the current setting. Similar to germ cell differentiation from hESCs^34^, priming of the naive cells to an epiblast-like pluripotency state maybe required. However, further studies are warranted to test whether this epiblast-like state is the equivalent or better than the primed counterpart. It also has to be taken into account that currently used differentiation protocols have been optimized using primed hESC, which mostly differ from the protocols used for directed differentiation in mESC. Hence, these results suggest that the current well-established differentiation protocols for primed hESCs need optimization when applied to naive hESCs to obtain mature cell types.

### Naive hESCs exhibit unique transcriptional profile

**Figure 5:**
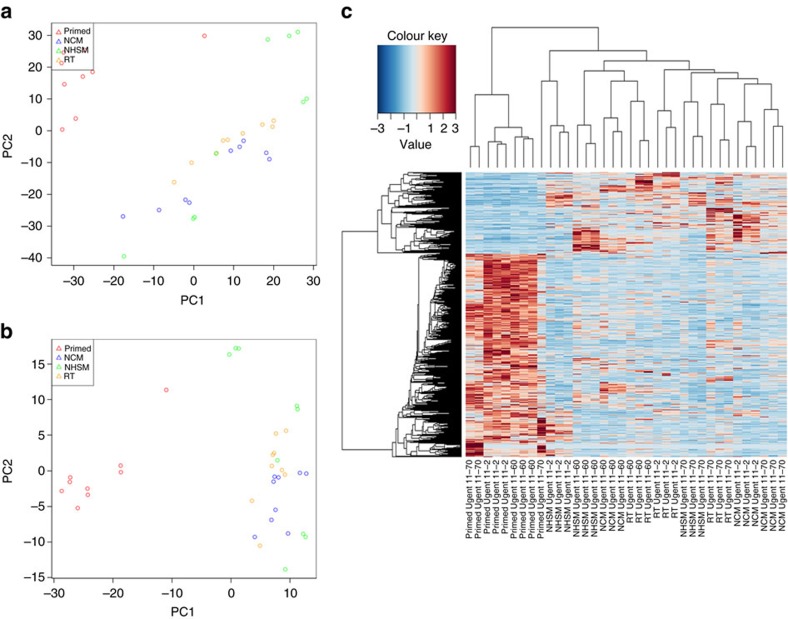
Genome-wide expression analysis of parental primed hESC and converted naive hESC in different conversion conditions. (**a**) PCA based on normalized transciptome data. (**b**) PCA based on rlog values of the differentially expressed genes (FC> 2, FDR<0.05) between primed hESC and all naive hESC. (**c**) Hierarchical clustering and heatmap of differentially expressed transcriptome data between parental primed and all naive hESCs.

Naive pluripotency-associated transcriptomic reconfiguration was determined using RNA-seq by comparing multiple and independent cultures of three primed hESC lines and their naive counterparts. A first principal component analysis (PCA) on normalized data showed that the primed cells separated from the converted naive hESC cells (Fig. 5a, Supplementary Data 1). Statistical differential expression analysis comparing primed hESC (selected as the control group) to all the naive converted cells revealed that ∼1,638 genes (false discovery rate (FDR)<0.05) were differentially expressed between the naive and primed hESCs. PCA and clustering analysis based on the differentially expressed genes separated all naive hESCs cultured in NCM-, NHSM- and RT-MEF naive media and exhibited a unique transcriptome compared to primed hESCs (Fig. 5b,c, Supplementary Data 2). GO analysis showed enrichment for terms related to RNA processing, DNA replication and repair, regulation of cell cycle process and downregulation of terms related to extra cellular adhesion and various developmental processes (Supplementary Data 3,4). Differential KEGG pathway analysis showed upregulation of pathways related to oxidative phosphorylation, cell cycle regulation whereas pathways related to differentiation were downregulated in the naive hESC (Supplementary Data 5). During human embryo development, there is an increase in dependence on oxidative phosphorylation as glucose can negatively affect early embryogenesis, and the pre-implantation blastocyst relies on this metabolic state facilitated by mitochondrial biogenesis and maturation^35^. Hence, the dependence of naive hESCs on oxidative phosphorylation instead of glycolytic metabolism, similar to human pre-implantation epiblast, is an important hallmark indicative of their naive state^35^,^36^,^37^. Next, to understand the differences between primed parental hESCs and the individual naive conversion conditions, differential expression was analysed between the primed hESC and the individual treatment groups. This analysis revealed 2,019, 1,996 and 2,007 genes to be differentially expressed when NCM, NHSM and RT conditions were individually compared to the primed condition, respectively (Supplementary Data 6–8). Gene set enrichment analysis showed upregulation of categories related to embryonic stem cell core and stemness in all naive hESCs. Interestingly, categories related to mitochondria, MYC targets and TGFβ were upregulated in NCM and NHSM conditions only (Supplementary Data 9–11).

**Figure 6:**
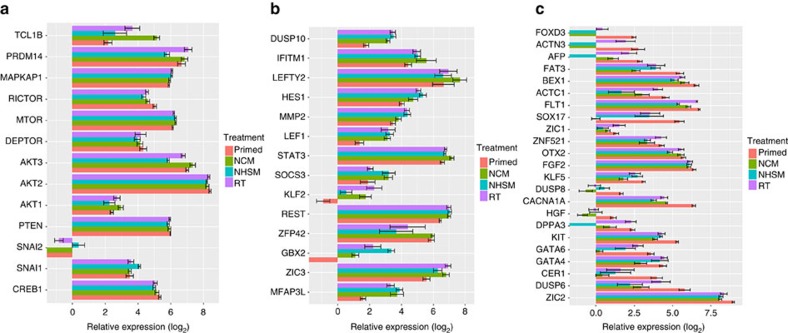
Panel of genes of interest. (**a**) Genes involved in PI3K/AKT/mTORC pathway. (**b**) Genes related to naive pluripotency upregulated in converted naive hESCS. (**c**) Genes related to lineage specification downregulated in converted naive hESCs. The data are represented as mean±s.e.m.

In-depth dissection of the data for a subset of key genes required for acquisition of naive pluripotency led to interesting observations. *AKT1* (also known as protein kinase B), is required by the PI3K/AKT pathway to facilitate cell-cell signalling via insulin-like growth factor, which leads to activation of the mTOR and inhibition of the GSK3β pathway^24^. We observed upregulation of *AKT1* in the naive hESCs (specifically in NCM-MEF, *n*=3, *P*<0.05), while it continued to be lowly expressed in the primed and NHSM-MEF naive hESCs (Fig. 6a). Increased *AKT1* phosphorylation is facilitated by the mTORC2 subunit, hence promoting cell proliferation and growth. *PTEN*, required in maintaining the negative feedback loop mechanism in PI3K/AKT pathway was expressed in all hESCs^38^. *RICTOR* and *MAPKAP1*, known genes exclusive to mTORC2 subunit^39^ showed increased expression compared to *DEPTOR*, a common subunit between mTORC1 and mTORC2 subunits, in the naive lines. Although primed hESCs exhibited a similar expression profile for *PTEN*, *mTOR*, *RICTOR, MAPKAP1* and *DEPTOR*, elevated expression of *PRDM14* (NCM- and RT-MEF), *TCL1B* (all naive hESC) and *AKT1* (NCM- and RT-MEF) in naive hESCs shows their increased dependence on PI3K/AKT/mTORC pathway^19^,^24^, specifically the mTORC2 subunit, as further validated by our inhibitor assay. SNAI1, although an epithelial-to-mesenchymal transition factor, is known to enhance reprogramming in human and mouse somatic cells when overexpressed^40^. Interestingly, we observed SNAI1 expression in naive hESCs increased, specifically in NHSM-MEF, compared to primed hESCs (Fig. 6a). Naive pluripotency markers including *LEF1*, *KLF2*, *ZIC3*, *LEFTY2*, *STAT3*, *HES1*, *SOCS3*, *MMP2, GBX2* were significantly increased (*P*<0.05) in all naive hESCs (Fig. 6b) whereas more primed pluripotency-associated genes such as *DUSP8*, *CACNA1A*, *HGF*, *CER1, FGF2* continued to be significantly downregulated^8^,^10^,^21^,^41^ (Fig. 6c). Naive hESCs, known to exist in a hypomethylated state^42^, showed a similar expression of *TET* enzymes compared to primed hESCs, however, all naive hESCs showed decreased levels of *de novo* methyltransferase *DNMT3A*, *DNMT3B* compared to primed hESCs whereas *DNMT3L* was significantly increased, similar to as observed in reset cells^13^ (Supplementary Data 6–8). Comparison of the different naive conversion conditions with each other (*F*-test, ANOVA, FDR<0.05) 4,195 genes were differentially expressed (Supplementary Fig. 9, Supplementary Data 12), such as naive markers including *GBX2, KLF2, DPPA3, SOAT1, PRDM14, TCL1B, SALL1* and differentiation-associated genes such as *GATA4, GATA6, AFP, SOX17, OTX2* between the three naive culture conditions.

Overall, we report an unbiased molecular and transcriptional analysis of naive hESCs generated in different culture conditions. Specifically, in-house-derived primed hESCs were converted in the same laboratory, unlike previous studies that conducted comparative analysis exclusively on published data sets. We also demonstrate that although the naive hESCs can differentiate efficiently towards immature cell types, the current differentiation protocols shown to be working efficiently for primed hESCs, probably require further optimization in order to be used for naive hESCs, as these two pluripotency states reflect the different stages of embryo development. Alternatively, it could be possible that naive pluripotent stem cells require a priming towards a more differentiated stage, before they acquire the full potential of efficient differentiation. Importantly, we confirm the role of PI3K/AKT/mTORC signalling in facilitating the induction of naive pluripotency in not only primed hESCs converted in NCM-MEF conditions, but also in NHSM- and RT-MEF naive hESCs.

## Methods

### Culturing of primed hESCs

In-house-derived primed hESC lines UGent11-2 (XX), UGent11-60 (XX) and UGent11-70 (XY)^8^ were maintained on mitomycin C treated-mitotically inactivated MEFs in low oxygen conditions (5%). The primed cell cultures were fed every day with fresh hESC medium composed of knockout-DMEM, 20% knockout serum replacement, 1% non-essential amino acids (NEAA), 1% l-glutamine, 1% penicillin/streptomycin, 0.1 mM β-mercaptoethanol (Invitrogen) and recombinant human basic fibroblast growth factor (bFGF, 4 ng ml^−1^, Peprotech). Confluent cell cultures (usually 5 days) were passaged using collagenase (1 mg ml^−1^, Invitrogen) and were split at a ratio of 1:2 to 1:4.

### Conversion of primed hESCs to a naive state in different naive media

Three primed hESC lines were subjected to naive conversion on MEFs in low oxygen conditions in either: (i) naive human stem cell medium (NHSM) composed of basal hESC media supplemented with AlbumaxI, N2 supplement (Invitrogen), PD0325901 (1 μM, Cayman) and CHIR99021 (3 μM, Axon Medchem; known together as 2i), leukaemia inhibitory factor (LIF, 1000U, Sigma), bFGF, (8 ng ml^−1^), SP600125 (10 μM, Tocris), SB203580 (10 μM, Tocris) and TGFβ (1 ng ml^−1^, Peprotech)^10^; (ii) RT consisting of high glucose DMEM-F12 (Invitrogen) further supplemented with histone deacetylase inhibitors, sodium butyrate (0.1 mM) and SAHA (50 nM) for first three passages followed by 2i and bFGF (10 ng ml^−1^)^11^ or (iii) our novel medium naive conversion medium (NCM) containing hESC media with added 2i, LIF, forskolin (10 μM, Sigma), ascorbic acid (50 ng ml^−1^, Sigma) and bFGF(12 ng ml^−1^)^8^. Upon the appearance of domed-shaped colonies, the culture was passaged using 0.05% trypsin and once the culture was well established, cells were split at a ratio of 1:4 to 1:8 every 3 days. Single-cell clonogenicity was determined from the number of single-cells plated (upon dissociating primed and naive hESCs using trypsin without ROCKi) and the subsequent number of colonies formed. Doubling time was calculated using the formula:


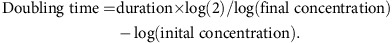


### Immunocytochemistry

Undifferentiated naive hESCs in the different media were fixed using 4% paraformaldehyde at room temperature (rt) for 20 min or at 4 °C overnight (O/N). The cells were then permeablized with 0.1% Triton X-100 in 1 × phosphate buffered saline (PBS) for 8 min, washed for 5 min in 1 × PBS and blocked in 1% bovine serum albumin in 1 × PBS for 1 h at rt. Primary antibodies, anti-goat OCT4 (Santa Cruz Biotechnology), anti-rabbit NANOG (R&D Systems), anti-rabbit PAX6 (Abcam), anti-mouse NKX2.5 (Abcam), anti-rabbit GATA4 (Santa Cruz Biotechnology), anti-mouse β-TUBULIN (Sigma-Aldrich), anti-rabbit TROPONIN (Santa Cruz Biotechnology) and anti-mouse MYOSIN (Chemicon) were diluted in the blocking solution (1:200) and the cells were then incubated with primary antibodies at 4 °C O/N. Next day, the cells were washed with 1 × PBS three times for 5 min and were incubated with secondary antibodies (diluted in blocking solution) for 1 h at rt. The secondary antibodies applied were donkey anti-goat FITC (1:200, Bioconnect), donkey anti-mouse Alexa 488 (IgG H&L, Life Technologies) and donkey anti-rabbit CY3 (1:200, Bioconnect). Finally, the cells were rinsed again in 1 × PBS three times for 5 min and mounted onto glass slides in Vectashield with 4',6-diamidino-2-phenylindole (DAPI).

### Differentiation assays

Spontaneous, directed, neuronal and cardiac differentiation assays were carried out as previously described^8^,^32^,^33^ in low oxygen conditions. In brief, for neuronal differentiation, cells were maintained on MEFs until they reached ∼80% confluency and were passaged to form EBs in 390 ml DMEM/F12, 5 ml NEAA, 5 ml 100 × GlutaMAX, 36 μl 2-mercaptoethanol and 100 ml KOSR (Invitrogen). On day 4, the media was changed to neural induction medium (NIM) composed of 490 ml DMEM/F12, 5 ml NEAA, 5 ml N2 supplement (Invitrogen) and 20 mg ml^−1^ Heparin (Sigma). On day 7, EBs were plated in NIM containing 5% fetal bovine serum to promote attachment for 8 h and were refreshed gently with fresh NIM once the EBs were attached. Neural rosettes were observed between days 10–12. For cardiomyocyte differentiation, cells were plated as single cells in the presence of ROCKi (Enzo Life) on matrigel (Corning). Differentiation was initiated once the cells reached 80–90% confluency and were cultured in RPMI/B27 (without insulin, Invitrogen) in the presence of CHIR9902 (12 μM, Axon MedChem) for 24 h. Next, the media was changed to only RPMI/B27 (without insulin) and 72 h later, half the media was refreshed with additional supplementation WNT inhibitor IWP2 (5 μM, Sigma) in the media. On day 5, the cells were refreshed with fresh RPMI/B27 (without insulin) medium and day 7 onwards, the cells were refreshed with RPMI/B27 (with insulin). Spontaneous contractions were observed between days 10–12. For spontaneous differentiation, primed, as well as different, naive hESCs were trypsinized and re-suspended in differentiation medium (DFM) similar to hESC medium without bFGF and the knockout serum replacement replaced with fetal bovine serum and cultured as EBs on 24-well Costar ultra-low attachment plates (Corning) for 14 days with medium changed every other day. For lineage-specific differentiation, cells were either exposed to DFM with 10 μM retinoic acid for ectodermal differentiation for 10 days; to DFM supplemented with 5 ng ml^−1^ bFGF and 10 mM nicotinamide (Sigma) for mesoderm formation for 8 days; or to DFM consisting of Activin A (20 ng ml^−1^, R&D Systems) and BMP4 (50 ng ml^−1^, R&D Systems) for an additional 7 days.

### Quantitative real-time PCR

RNA isolation, cDNA synthesis, quantitative real-time PCR (qRT-PCR) gene expression and data analysis was performed as previously described^8^ for primed hESCs, EBs and naive hESCs and EBs. Briefly, RNA isolation and cDNA synthesis was performed using RNeasy Mini Kit (Qiagen) and iSCRIPT Advanced cDNA synthesis Kit (Biorad), respectively, and the cDNA concentration was determined using Qubit fluorometric quantitation method (Thermofisher Scientific). For subsequent qRT-PCR gene expression analysis, 10 ng cDNA was used for each reaction per gene, performed in technical and biological replicates on an ABI 7000 sequence detection system (Applied Biosystems). Housekeeping genes included *GAPDH*, *B2M* and *RPL13A*, naive pluripotency markers used were *KLF2*, *KLF4*, *ESRRB*, *PRDM14*, *REX1*, *TCL1B, OCT4* and *NANOG*. Germ layer-specific markers used were *NESTIN* and *PAX6* (ectoderm), *T-BRACHYURY, HAND1* and *PECAM1* (mesoderm), *AFP*, *GATA4* and *GATA6* (endoderm). CT values were normalized to housekeeping genes and fold change was analysed using the ΔΔCT method where all samples were compared to their respective controls in each experiment. Statistical significance was calculated using the ANOVA and *t*-test on fold change gene expression values (2^−ΔΔCT^). No RT (only RNA) or no template (water) were used as negative controls.

### Array-based comparative genome hybridization

Whole-genome amplification of the samples was performed using the SurePlex Amplification system following manufacturer's instructions (Illumina Bluegnome, United Kingdom). A total of 2.5 μl of control DNA (G1521; Promega; 187 ng μl^−1^) was used at a concentration of 25 pg μl^−1^. Whole-genome amplification products were hybridized on 24sure+ arrays (Illumina Bluegnome, UK) according to manufacturer's instructions. A male reference sample was used for all tested samples. Briefly, amplified samples (from one colony/naive condition at passage 48) and reference DNA were labelled with Cy3 and Cy5 fluorophores, respectively, for 4 h at 37 °C. After combining sample and reference with Cot1 DNA, the mixture was precipitated for 20 min at −80 °C. The pellets were re-suspended in pre-warmed hybridization buffer and hybridized on a 24sure+ array for 16 h (overnight) at 42 °C. After washing, microarrays were scanned on an Agilent G2565CA microarray scanner (Agilent, USA). Data were analysed using the BlueFuse Multi software (Illumina Bluegnome, United Kingdom) and visualized in the Vivar software.

### RNA-seq method and data analysis

Total RNA was extracted using Qiagen RNeasy mini kit, according to the manufacturers' protocol, including a DNAse digestion. Concentration and quality of the total extracted RNA was checked by using the `Quant-it ribogreen RNA assay' (Life Technologies, Grand Island, NY, USA) and the RNA 6000 nano chip (Agilent Technologies, Santa Clara, CA, USA), respectively. Subsequently, 500 ng of RNA was used to perform an Illumina sequencing library preparation using the QuantSeq 3' mRNA-Seq Library Prep Kits (Lexogen, Vienna, Austria) according to manufacturer's protocol. Libraries were quantified by qPCR, according to Illumina's protocol `Sequencing Library qPCR Quantification protocol guide', version February 2011. A High sensitivity DNA chip (Agilent Technologies, Santa Clara, CA, USA) was used to control the library's size distribution and quality. Sequencing was performed on a high throughput Illumina NextSeq 500 flow cell generating 75 bp single reads.

Per sample, on average 5.8 × 10^6^±1.5 × 10^6^ reads were generated. First, these reads were trimmed using cutadapt^43^ version 1.11 to remove the ‘QuantSEQ FWD' adaptor sequence. To remove contamination with mouse DNA, the reads were mapped against the Mus musculus GRCm38.p5 reference genome using CLC genomics workbench version 9.0.1 (CLC). Only the reads that didn't map against the GRCm38.p5 genome were subsequently mapped against the Homo sapiens hg19 GRCH37.74 genome using CLC. Count tables were made by counting the reads that mapped against the genes defined by the Ensembl GRCh37.74 Gene transfer format file and exported from CLC.

To explore if the samples from different treatment groups clustered together and to detect outlier samples, a PCA was performed in R on the rlog^44^ normalized and transformed counts of the genes expressed at c.p.m. above 1 in at least two samples.

Differential gene expression analysis was performed using edgeR^45^. Genes were only retained if they were expressed at a c.p.m. above 1 in at least two samples. In one differential gene expression analysis, a general linear model was made with two treatments ‘primed' versus ‘non-primed' (including the ‘NCM', ‘NHSM' and ‘RT' samples), defining the effects from the three cell lines ‘UGent11-60', ‘UGent11-2' and ‘UGent11-70' as batch effect. Statistical testing was done using the empirical Bayes quasi-likelihood *F*-test. Genes having a FDR<0.05 and a fold change>2 were considered significantly differential. PCA was performed in R on rlog^44^ transformed c.p.m. of the differentially expressed genes. To produce the heat map, the normalized count data of the differentially expressed genes was rescaled between −3 and 3 and clustered based on Pearson correlation^46^. In an alternative differential gene expression analysis, a general linear model was made with four treatments ‘primed', ‘NCM', ‘NHSM' and ‘RT', defining the effects from the three cell lines ‘UGent11-60', ‘UGent11-2' and ‘UGent11-70' as batch effect. The latter model was used to statistically compare ‘primed' versus ‘NCM', ‘primed' versus ‘NHSM' and ‘primed' versus ‘RT'. Statistical testing was done using the Bayes quasi-likelihood *F*-test. Genes with an FDR<0.05 and a fold change>2 were considered significantly differential. To compare the three ‘non-primed' treatments, a general linear model was made with three treatments ‘NCM', ‘NHSM' and ‘RT', defining the effects from the three cell lines ‘UGent11-60', ‘UGent11-2' and ‘UGent11-70' as batch effect. The latter model was used to statistically test any difference between the three ‘non-primed' treatments using empirical Bayes quasi-likelihood *F*-test. Genes with a FDR<0.05 were considered significantly differential. PCA was performed in R on rlog^44^ transformed c.p.m. of the differentially expressed genes. To produce the heat map, the normalized count data of the differentially expressed genes was rescaled between −3 and 3 and clustered based on Pearson correlation^46^.

Differential expression of pathways in the gene set data collection ‘kegg.gs' derived from the KEGG database, was based on log fold changes of the pairwise comparison of ‘primed' versus ‘NCM', ‘primed' versus ‘NHSM' and ‘primed' versus ‘RT' using the GAGE R package^47^. Pathways were considered differentially expressed when the average log fold change of the pathway was significantly different from the overall average log fold change at an FDR<0.05. Differentially expressed pathways were visualized using Pathview^48^, showing the log fold change for each comparison and each enzyme in the pathway. Analogously, a differentially expression analysis was performed on the biological and the molecular function GO-terms and the chemical and genetic perturbations gene set from the Molecular Signatures Database^49^. Gene sets were considered differentially expressed when the average log fold change of the gene set was significantly different from the overall average log fold change at an FDR<0.05.

### Data availability

The authors declare that all supporting data are submitted under BioSample accession numbers SAMN06111297, SAMN06111298, SAMN06111299, SAMN06111300, SAMN06111301, SAMN06111302, SAMN06111303, SAMN06111304, SAMN06111305, SAMN06111306, SAMN06111307, SAMN06111308 and also supplied as Supplementary Datasets.

## Additional information

**How to cite this article:** Warrier, S. *et al*. Direct comparison of distinct naive pluripotent states in human embryonic stem cells. *Nat. Commun.* **8,** 15055 doi: 10.1038/ncomms15055 (2017).

**Publisher's note:** Springer Nature remains neutral with regard to jurisdictional claims in published maps and institutional affiliations.

## Supplementary Material

Supplementary Information Supplementary Figures

Supplementary Data 1 Normalised Count Table

Supplementary Data 2 Differential Gene Expression Analysis between primed hESC and all naïve converted hESC (FC>2, FDR<0.05)

Supplementary Data 3 Biological Processes GO (Gene Ontology) Terms for comparing all naïve hESC to the primed counterparts.

Supplementary Data 4 Molecular Functions GO (Gene Ontology) Terms for comparing all naïve hESC to the primed counterparts.

Supplementary Data 5 KEGG (Kyoto Encyclopedia of Genes and Genomes) Pathways enriched for naïve hESC as compared to their primed counterparts.

Supplementary Data 6 Differential Gene Expression Analysis between primed hESC and NCM-MEFs hESCs (FC>2, FDR<0.05)

Supplementary Data 7 Differential Gene Expression Analysis between primed hESC and NHSM-MEFs hESCs (FC>2, FDR<0.05)

Supplementary Data 8 Differential Gene Expression Analysis between primed hESC and RT-MEFs hESCs (FC>2, FDR<0.05)

Supplementary Data 9 GSEA (Gene Set Enrichment Analysis; CGP, Chemical and Genetic purtabation gene sets) between NCM-Mefs converted hESCs and primed hESCs.

Supplementary Data 10 GSEA (Gene Set Enrichment Analysis; CGP, Chemical and Genetic purtabation gene sets) between NHSM-Mefs converted hESCs and primed hESCs.

Supplementary Data 11 GSEA (Gene Set Enrichment Analysis; CGP, Chemical and Genetic purtabation gene sets) between RT-Mefs converted hESCs and primed hESCs.

Supplementary Data 12 Differential Gene Expression Analysis between the three naïve conversion conditions (ANOVA-like test, FDR<0.05)
