# Supplementary material for: Direct comparison of distinct naive pluripotent states in human embryonic stem cells
Source: Nat Commun. 2017 Apr 21;8:15055. doi: 10.1038/ncomms15055 (PMC5413953; doi:10.1038/ncomms15055)
Supplement: Supplementary Information — Supplementary Figures [file ncomms15055-s1.pdf]

Supplementary Figure 1

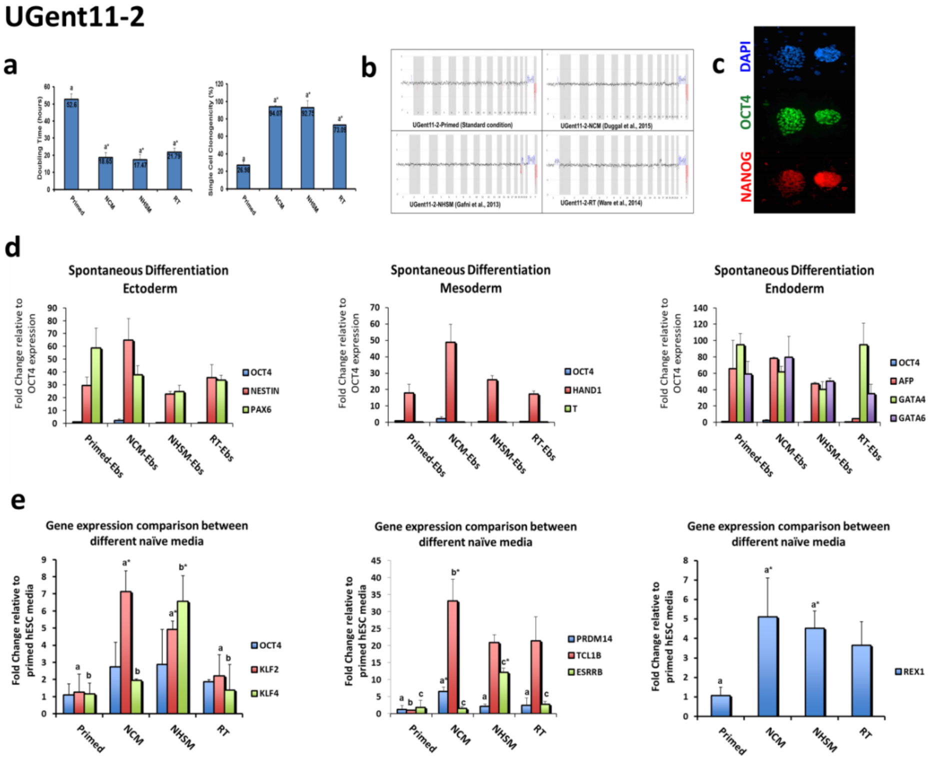

Supplementary Figure 1: Assessment of naïve pluripotency in the newly formed naïve hESCs from in-house derived, established primed hESC line UGent11-2. **a,** Doubling time and single cell clonogenicity assays for primed hESCs, NCM-, NHSM- and RT-naïve hESCs. a* compared to a and b* compared to b, * represents $p<0.05$. **b,** Depiction of chromosomal analysis for primed hESCs, NCM-, NHSM- and RT-naïve hESCs via aCGH. **c,** Representative immunofluorescence image for pluripotency makers OCT4 and NANOG in NCM-, NHSM- and RT-naïve hESCs; Scale bar, 200µm. **d,** qRT-PCR analysis for ectoderm, mesoderm and endoderm markers on spontaneously differentiated primed hESCs, NCM-, NHSM- and RT-naïve hESCs as EBs for 14 days. **e,** Gene expression analysis for naïve-pluripotency specific genes on undifferentiated primed hESCs, NCM-, NHSM- and RT-naïve hESCs. a* compared to a and b* compared to b, * represents $p<0.05$.

Supplementary Figure 2

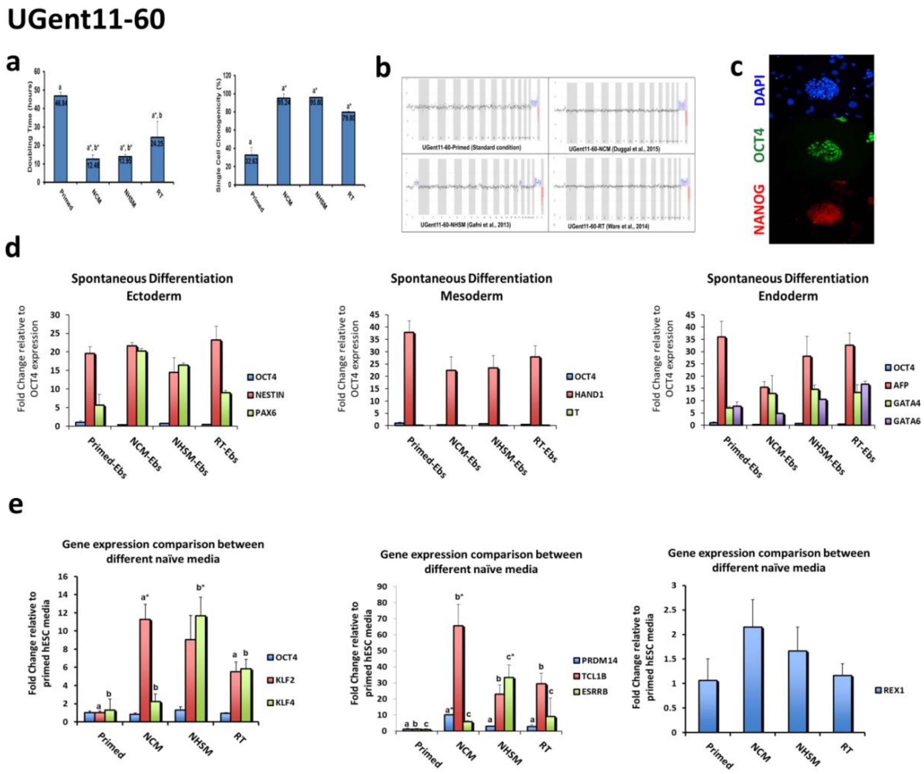

Supplementary Figure 2: Assessment of naïve pluripotency in the newly formed naïve hESCs from in-house derived, established primed hESC line UGent11-60. **a,** Doubling time and single cell clonogenicity assays for primed hESCs, NCM-, NHSM- and RT-naïve hESCs. a* compared to a and b* compared to b, * represents p<0.05. **b,** Depiction of chromosomal analysis for primed hESCs, NCM-, NHSM- and RT-naïve hESCs via aCGH. **c,** Representative immunofluorescence image for pluripotency makers OCT4 and NANOG in NCM-, NHSM- and RT-naïve hESCs; Scale bar, 200µm. **d,** qRT-PCR analysis for ectoderm, mesoderm and endoderm markers on spontaneously differentiated primed hESCs, NCM-, NHSM- and RT-naïve hESCs as EBs for 14 days. **e,** Gene expression analysis for naïve-pluripotency specific genes on undifferentiated primed hESCs, NCM-, NHSM- and RT-naïve hESCs. a* compared to a and b* compared to b, * represents p<0.05.

Supplementary Figure 3

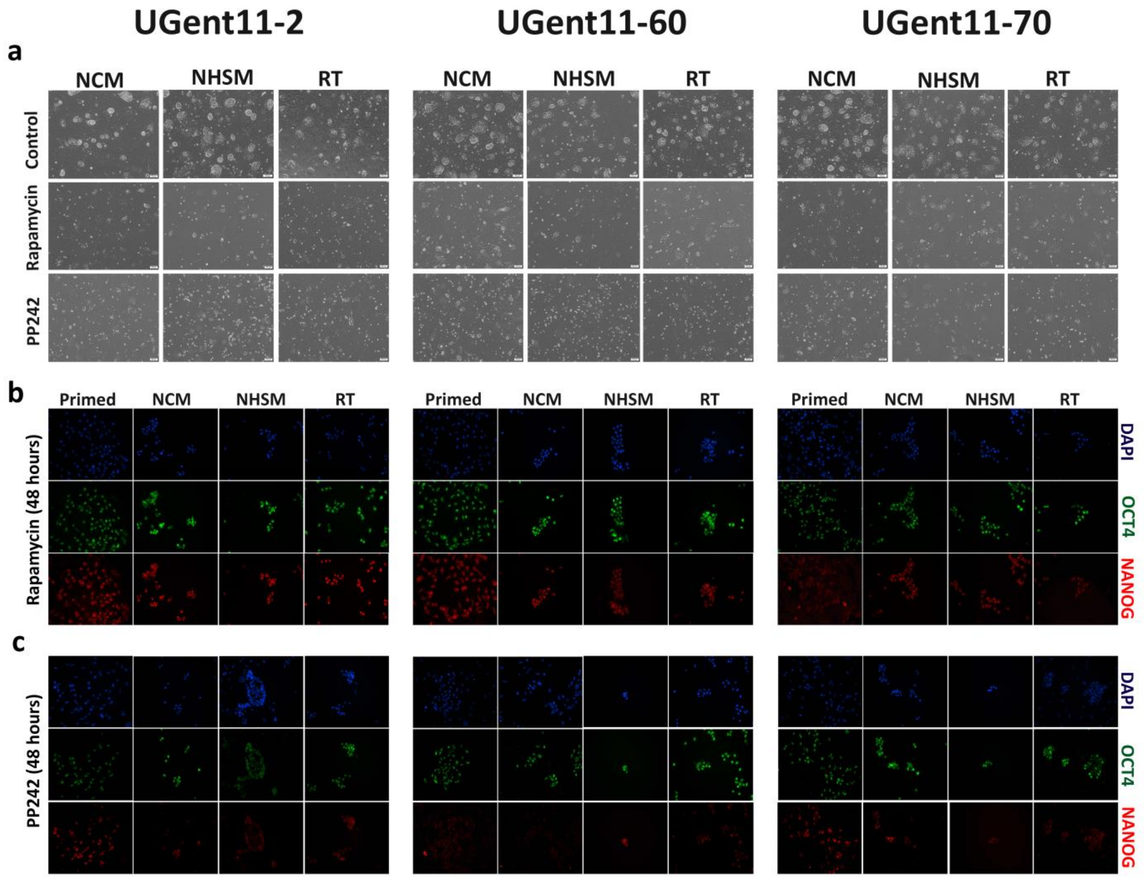

Supplementary Figure 3: Mechanistic assay determining the dependence of primed and naïve hESCs on PI3K/AKT/mTORC pathway. **a**, 48 hour exposure to mTORC1 inhibitor Rapamycin and mTORC1/2 inhibitor PP242 revealed increased cell death in all naïve hESCs compared to primed hESCs (increased number of floating dead cells observed upon exposure to PP242). Scale bar, 200µm. **b** and **c**, Immunofluorescence images for pluripotency markers OCT4 and NANOG decreased expression in all naïve hESCs, with NANOG expression completely lost upon exposure to PP242.). Scale bar, 200µm.

Supplementary Figure 4

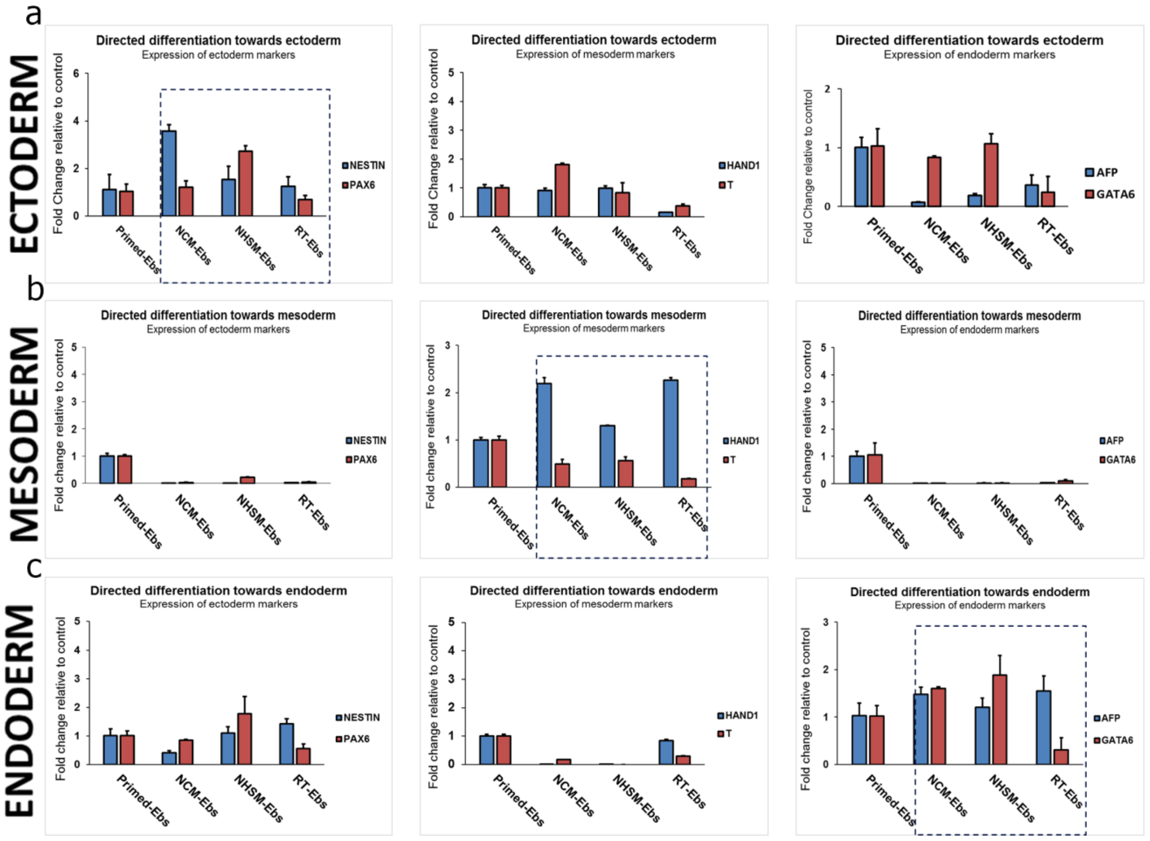

Supplementary Figure 4: Gene expression analysis for lineage-specific genes upon directed differentiation of naïve hESC line UGent11-2 towards **a,** ectoderm, **b,** mesoderm and **c,** endoderm. All germ layer markers were analysed irrespective of the targeted lineage to determine the heterogeneity in differentiation between the different naïve medium conditions as well as between primed and naïve conditions. Dashed box represents the gene expression for markers specific for that lineage.

Supplementary Figure 5

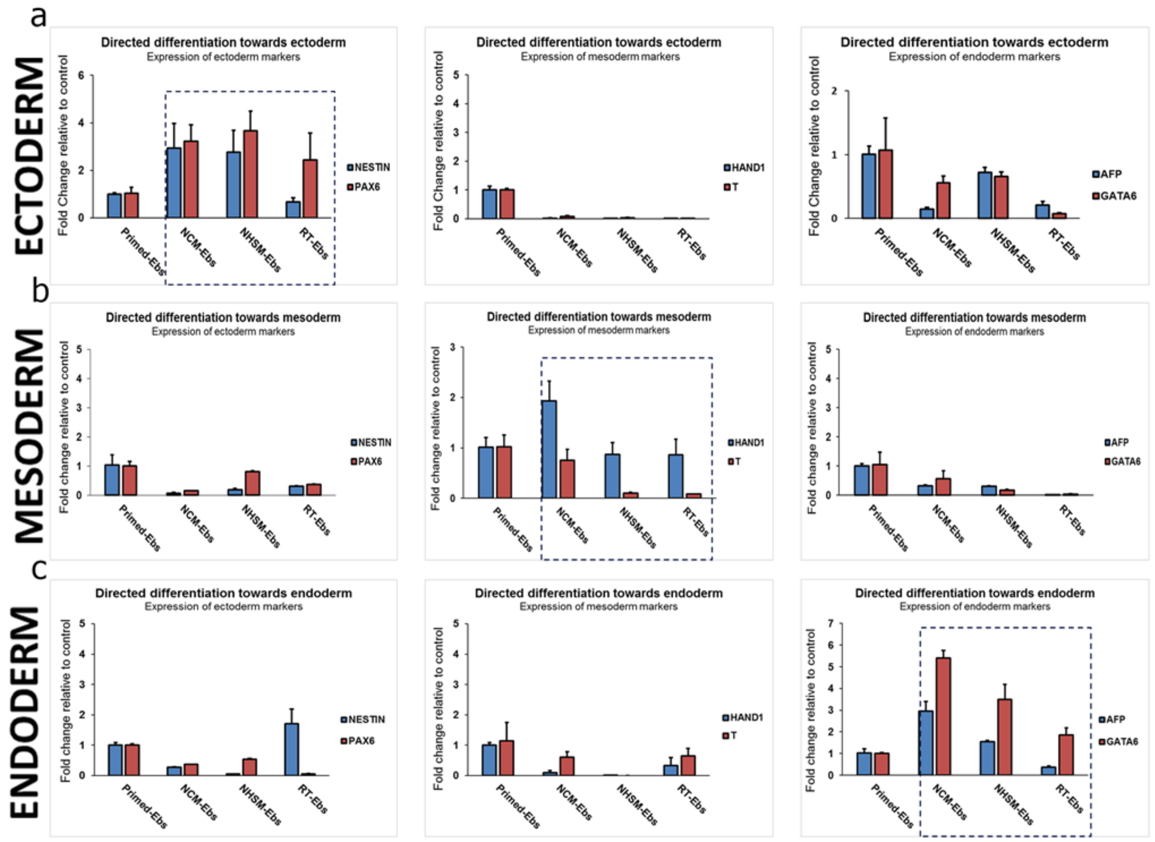

Supplementary Figure 5: Gene expression analysis for lineage-specific genes upon directed differentiation of naïve hESC line UGent11-60 towards **a,** ectoderm, **b,** mesoderm and **c,** endoderm. All germ layer markers were analysed irrespective of the targeted lineage to determine the heterogeneity in differentiation between the different naïve medium conditions as well as between primed and naïve conditions. Dashed box represents the gene expression for markers specific for that lineage.

Supplementary Figure 6

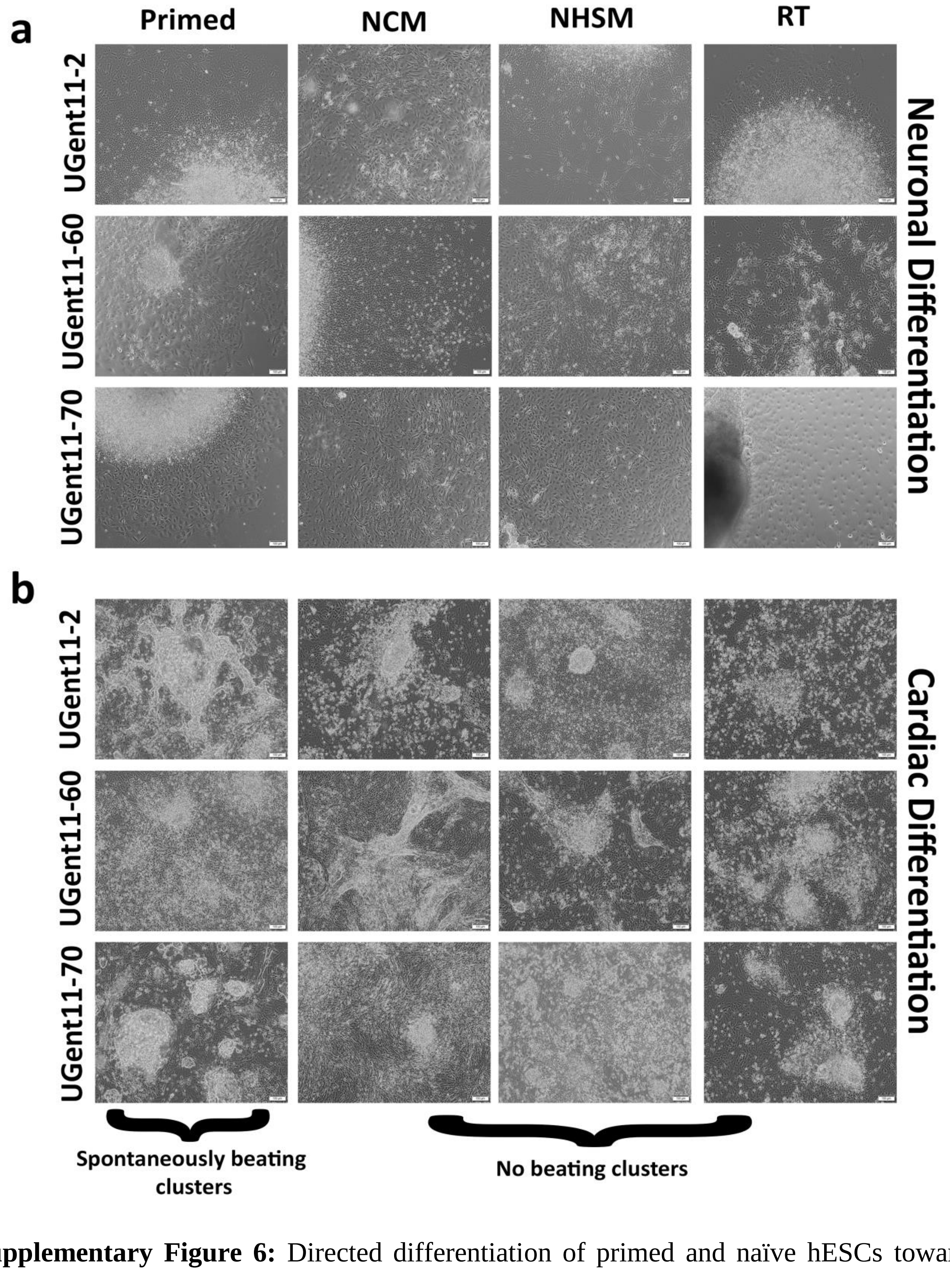

Supplementary Figure 6: Directed differentiation of primed and naïve hESCs towards **a,** Neuronal lineage and **b**, Cardiac lineage. Although all primed and naïve hESCs formed neuronal processes, however upon differentiation towards cardiomyocytes, only those derived from primed hESCs developed beating clusters. Scale bar, 200µm.

Supplementary Figure 7

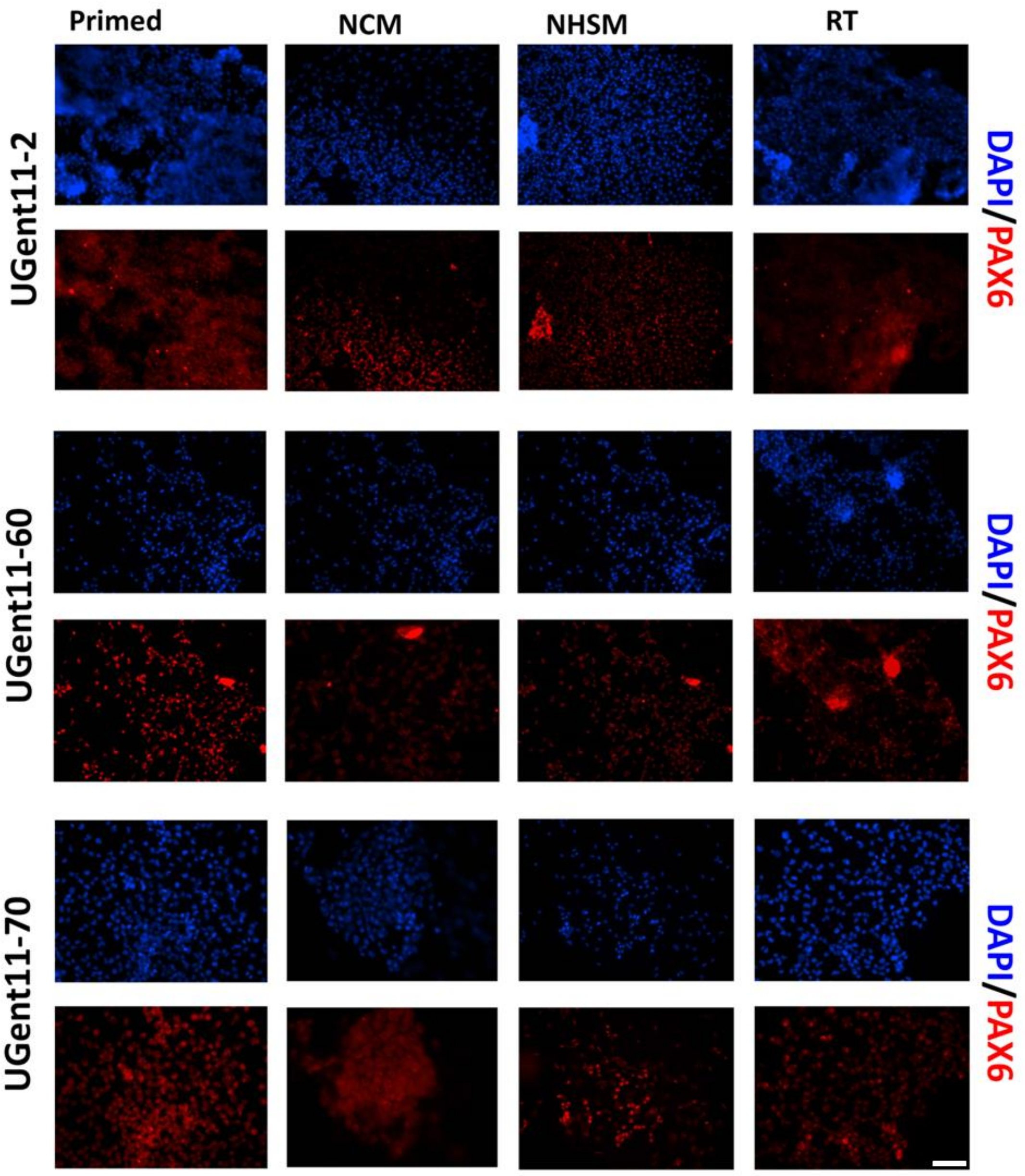

Supplementary Figure 7: Immunofluorescence images demonstrate all primed and naïve hESCs express neuronal progenitor marker PAX6 upon targeted differentiation towards neurons. Scale bar, 200µm

Supplementary Figure 8

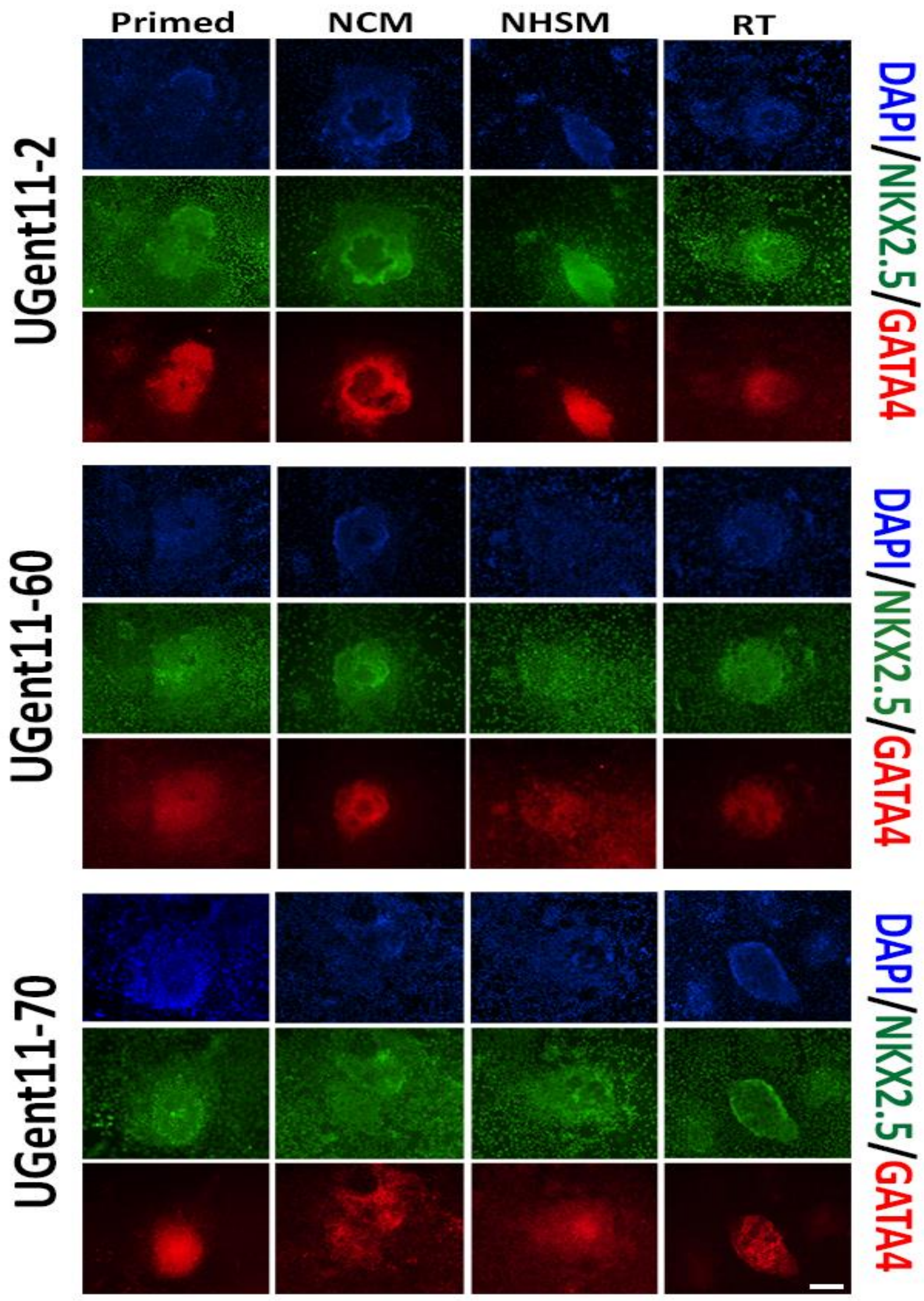

Supplementary Figure 8: Immunofluorescence images demonstrate all primed and naïve hESCs express cardiac progenitor markers NKX2.5 and GATA4 upon targeted differentiation towards cardiomyocytes. Scale bar, 200µm

Supplementary Figure 9

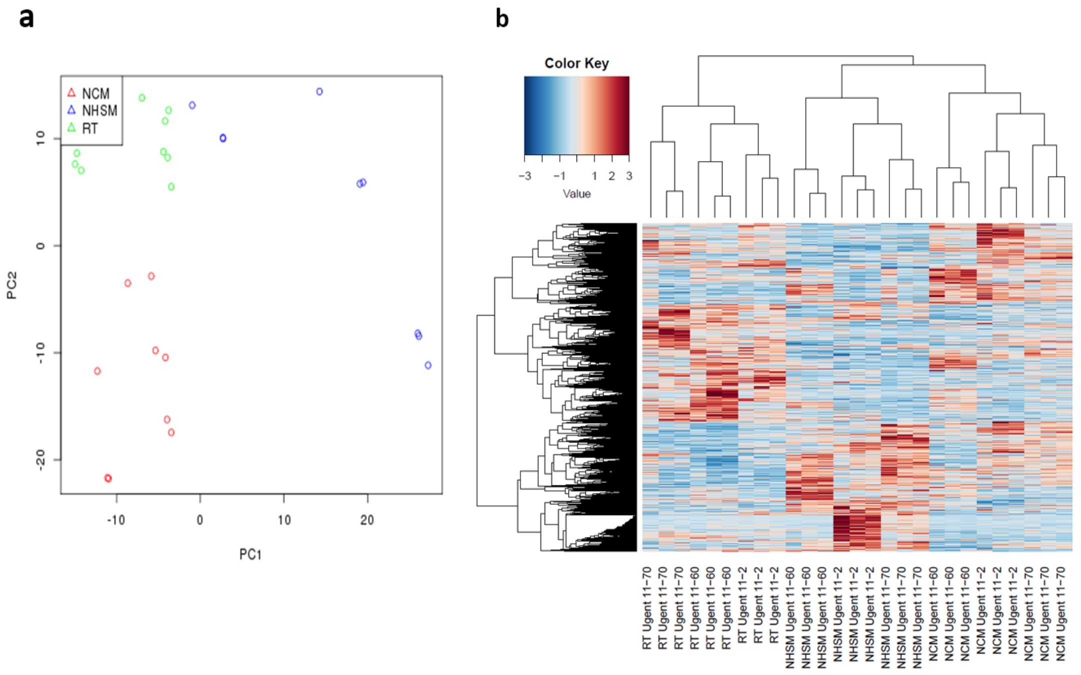

Supplementary Figure 9: Differential expression profiles of converted naïve hESCs. **a,** PCA representing rlog transformed counts of the differentially expressed genes between the three naïve conversion conditions (FDR<0.05). **b,** Hierarchical clustering and heatmap on normalised counts of differentially expressed genes between three naïve conversion conditions.
